# Amplitude-reduction alert criteria and intervention during complex paediatric cervical spine surgery

**DOI:** 10.1016/j.cnp.2022.07.003

**Published:** 2022-07-28

**Authors:** William M. McDevitt, Laura Quinn, W.S.B. Wimalachandra, Edmund Carver, Catalina Stendall, Guirish A. Solanki, Andrew Lawley

**Affiliations:** a Department of Neurophysiology, Birmingham Children’s Hospital, Birmingham, United Kingdom; b Institute of Applied Health Research, University of Birmingham, United Kingdom; c Birmingham Biomedical Research Centre, University Hospitals Birmingham NHS Foundation Trust and University of Birmingham, United Kingdom; d Department of Neurosurgery, Birmingham Children’s Hospital, Birmingham, United Kingdom; e Department of Anaesthesiology, Birmingham Children’s Hospital, Birmingham, United Kingdom

**Keywords:** Intraoperative neuromonitoring, Pediatric spinal surgery, Electrophysiology, Somatosensory evoked potential, Motor evoked potential, Neurosurgery

## Abstract

**Objective:**

To determine the utility of widely used intraoperative neuromonitoring (IONM) alert criteria and intervention for predicting postoperative outcome following paediatric spinal surgery.

**Methods:**

Retrospective analysis of somatosensory evoked potentials (SSEP) and motor evoked potentials (MEP) in consecutive cervical spine fixations. An intervention protocol followed amplitude-reductions in SSEPs (≥50 %) and/or MEPs (≥80 %). Alert breaches were reversed when SSEP/MEP amplitude was restored to > 50 %/20 % of baseline. Sensorimotor function was assessed preoperatively and 3-months postoperatively via the Modified McCormick Scale score (MMS). We explored associations between postoperative outcome, demographic/surgical and IONM variables.

**Results:**

Forty-five procedures in 38 children (mean age:9 ± 4 years;55 % female) were monitored, 42 %of which breached alert criteria. Instrumentation (6/19,32 %) and hypotension (5/19,26 %) were common causes for alert and the majority (13/19,68 %) were reversed following intervention. There was an association between pre- and post-MMS and the type of breach (p = 0.002). All children with worse postoperative MMS (3/38,8%) had irreversible breaches.

**Conclusions:**

IONM in this small sample accurately detected neurological injury. The majority of breaches reversed following an intervention protocol. Irreversible breaches frequently led to worse postoperative sensorimotor function.

**Significance:**

An intervention protocol which reversed IONM alerts never resulted in postoperative worsening of sensorimotor function.

## Highlights

• Alert criteria breaches occur frequently and are reversed following intervention during complex paediatric cervical spine surgery.
• All patients with worsening sensorimotor function had irreversible alert criteria breaches.
• Evoked potential amplitude reduction may provide an early warning to worsening sensorimotor function.

## 1 Introduction

Intraoperative neuromonitoring (IONM) of somatosensory evoked potentials (SSEP) and motor evoked potentials (MEP) during spinal surgery aims to prevent neurological injury by measuring amplitude change in comparison to a baseline recording (Dineen and Simon, 2018). When evoked potentials (EP) breach pre-defined alert criteria, the surgery is paused whilst attempts are made to re-establish the EP by optimising systemic, technical and surgical variables via an intervention protocol (Ziewacz et al., 2012, Vitale et al., 2014). If irreversible, postoperative deficit is frequent (Thirumala et al., 2016, Di Martino et al., 2019, Holdefer and Skinner, 2020).

However, the current evidence base for IONM is limited by few controlled trials, variation in MEP alert criteria, conflicting findings in the literature, and paradox or bias in the categorisation of recovered EPs (Howick et al., 2016, Skinner and Sala, 2017). These limitations make it difficult to build a consensus of what evidence is relevant when deciding the alert criteria to employ, and whether implementation of an intervention protocol improves outcome.

Controlled research in this area is challenging due to the ethical dilemma of performing IONM without acting on EP deterioration (Skinner and Holdefer, 2014). A common alternative is to report postoperative outcome of surgical cohorts where IONM was performed. If an irreversible breach predicts postoperative deficit and reversible breach predicts absence of postoperative deficit, IONM alert criteria and intervention is supported (Holdefer and Skinner, 2020).

This approach requires a definition of optimal alert criteria. The only major MEP IONM alert criterion for spinal surgery is absence of a previously present EP (Macdonald et al, 2013). This may limit the effect of an intervention protocol if irreversible neurological injury has already occurred, similarly true with SSEP absence. For this reason, reduction in amplitude criteria are often employed but this can be associated with false predictions if systemic and non-pathological factors causing EP deterioration are not taken into account.

Our aim was to determine the utility of widely used amplitude-reduction IONM alert criteria for predicting postoperative outcome following spinal surgery. We report a retrospective cohort of complex paediatric cervical spine cases performed with IONM at our institution.

## 2 Methods

### 2.1 Study design

A single centre retrospective analysis of consecutive paediatric patients requiring cervical spine fixation and/or decompression between March 2015 – February 2022 was performed. Ethical approval was granted by our institutions research and ethics department. Patient data was excluded if IONM was not utilised or provided by an external company, or if there was insufficient follow-up data.

A standardised protocol for IONM in spine procedures, including an intervention protocol following alert criteria breach was developed by one of the co-authors (GS) with input from anaesthesiology and neurophysiology. In brief, this provided an algorithm for IONM implementation in various situations; the methodology for preoperative neurophysiology, multidisciplinary team meeting, surgical team brief, and all pertinent intraoperative steps.

### 2.2 SSEP and MEP recordings

IONM of SSEPs and MEPs were performed using NIM Eclipse (Medtronic, Jacksonville, FL) software. General anaesthesia was induced either via inhaled agents, then switched to total intravenous propofol and remifentanil for maintenance, or total intravenous propofol and remifentanil from the start. Blockade of the neuromuscular junction (via rocuronium) was limited to anaesthetic induction and a train of four technique was utilised for the assessment of this. Intraoperative EPs were compared to a baseline obtained in the outpatient setting and adjusted following patient positioning and/or anaesthetic fade.

SSEPs were elicited via either median, ulnar, or tibial nerve stimulation at a rate between 2.1 and 5.1 Hz, each pulse with a 200-300µS duration. Responses were always recorded from the popliteal fossa and cortex (Cz’, Fz, C3′, C4′), and where appropriate the spinal column (cervical and/or thoracolumbar). These were recorded semi-continuously throughout the procedure and for at least 20 minutes post-procedure.

MEPs were elicited via a train of pulses (4–9) separated by a 2–4 ms interstimulus interval (ISI) using a constant voltage setup. The duration of each pulse was 50–75 µs. Muscles monitored were surgery dependant but included at least 2 upper limb and 2 lower limb muscles bilaterally (typically abductor hallucis, tibialis anterior, intrinsic hand, and deltoid muscles). Control MEPs (typically trapezius muscle) from above the site of surgery were recorded bilaterally where possible.

MEPs were acquired at 5 to 15-minute intervals; before and after major procedural steps (positioning, laminectomy/laminoplasty cuts, screw insertion, traction, distraction, spinal column manipulation) and at the discretion of the surgeon or IONM practitioner. Spontaneous electromyography was recorded from the muscles sampled for MEP monitoring. Persistent discharges prompted MEP acquisition. Detailed EP stimulation and recording parameters are provided as Supplementary Material.

### 2.3 Alert criteria and intervention protocol

IONM alerts were defined as a reduction in amplitude ≥ 50 % for SSEPs and ≥ 80 % for MEPs in line with previous publications (Nuwer et al., 1995, Langeloo et al., 2007). We considered 30–40 % SSEP decrements as an alert following the publication of IFCN standards (MacDonald et al, 2019). Alerts were classified as reversed if the SSEP/MEP amplitude was >50 %/>20 % of baseline. We did not elicit MEPs at supramaximal intensity in order to prevent excessive movement and were cognisant of the increased risk of false MEP alarms.

“Modified” alert criteria were employed when ≥50 % and ≥80 % amplitude decrements could not be determined. This occurred when EP amplitude was low at baseline; if there was high trial-to-trial amplitude variation and poor waveform superimposition for SSEPs; if there was high amplitude or morphology variation in MEPs. In these cases, the absence of a previously present EP was communicated as an alert.

In the event of an alert criteria breach, the IONM practitioner confirmed the observation with a second operator (either the clinical lead for IONM or a consultant in clinical neurophysiology). Following this, technical factors for alarm (which include cable integrity, equipment malfunction, and electrode impedance) were ruled out. This was followed by an assessment of breach laterality (ipsilateral vs bilateral) and severity (absent vs present but below threshold criteria) against a control EP where possible; and whether the change was identified in SSEP, MEP, or both modalities.

Following this, systemic and anaesthetic factors were assessed. In brief, this consisted of identifying whether the change coincided with a recent drug bolus; an acute shift in the depth of anaesthesia, heart rate, blood pressure, other vital signs and blood gas parameters. If the aforementioned were identified, they were corrected. If the alert occurred following prone positioning, the neck and torso were re-positioned, and the intervention protocol was only followed if the re-positioning did not reverse the alert. If the alert persisted, or was thought to be the result of a surgical event, the following intervention protocol was utilised.

Mean arterial pressure was increased by approximately 10 mmHg and propofol sedation was lightened if clinically indicated. Depth of anaesthesia was monitored using the Patient State Index (Sedline, Massimo, USA) or Bispectral Index (BIS, Covidien, USA) to ensure an appropriate level of anaesthesia was maintained. SSEP and MEP stimulation was increased by 10 mA and 100 V, respectively. For MEPs, the number of trains and pulse duration were increased where possible. Compound muscle action potentials (CMAPs) were facilitated via double train and/or rapid/consecutive acquisition, and the ISI was altered where appropriate. The surgeon reviewed whether neural tissue could have been compromised by screw insertion, traction, distraction, bleeding, heat (via drilling or electrocautery), compression, or cerebrospinal fluid blockage.

### 2.4 Data collection

Patient demographics, surgical, and IONM variables were collected from medical records. Primary outcome was Modified McCormick Scale score (MMS) (McCormick and Stein, 1990). This was assessed by the neurosurgical team preoperatively and at 3-month postoperative review (Table 1). Alert causation was categorised into correction (rod contouring, spine realignment, traction or distraction); exposure and instrumentation (screw, rod, plate, or retractor placement); hypotension; patient positioning; and unknown groups in a similar vein to previously published literature (Holdefer and Skinner, 2020).

**Table 1:** Modified McCormick Scale score (MMS) and description of category.

MMS	Description
1	Neurologically normal, ambulates normally, may have minimal dysaesthesia.
2	Mild motor or sensory deficit; independent function and ambulation maintained.
3	Moderate sensorimotor deficit, restriction of function, independent with external aid.
4	Severe motor or sensory deficit, restricted function, dependent.
5	Paraplegia or quadriplegia (even if there is flickering movement).

### 2.5 Statistical analysis.

Descriptive data were reported as frequencies and percentages for categorical variables, and either means with standard deviation (SD) or median with interquartile range (IQR) for continuous variables. A contingency table (3 × 3) with type of breach (no alert breach, reversed breach, and irreversible breach) and outcome (improved, stable or worse MMS) was created. Chi-square tests and Fisher’s exact test were used to test for associations between MMS, breach type and causation, demographic and surgical characteristics. Results were considered significant when *p* < 0.05. All data was analysed using Stata v16.1.

## 3 Results

Two hundred and eighty-eight consecutive paediatric neurosurgical procedures required IONM between March 2015-February 2022, of which 49 had cervical spine fixation and/or decompression. Exclusions were due to external IONM provision (*n* = 3) or death before 3-month postoperative assessment (*n* = 1). This resulted in a cohort of 38 paediatric patients (Mean age: 9 ± 4 years) and 45 procedures, as some required multiple operations. Patients considered neurologically normal, with normal ambulation and minimal dysaesthesia preoperatively (MMS: 1) were infrequent (14/45, 31 %). High-risk craniocervical junction (CCJ) fixation and/or decompression (35/45, 78 %) was monitored more frequently than sub-axial cervical spine procedures (Table 2).

**Table 2:** Demographic characteristics. Number and percentage reported unless stated otherwise. *Patients can have multiple procedures and multiple diagnoses within each subcategory.

Characteristic	N (%)
**Age, years - mean (SD)**	8.6 (4.2)
**Female**	21 (55)
Diagnosis*	
**Congenital disease**	23 (61)
Mucopolysaccharidosis	12 (52)
Neurofibromatosis	3 (13)
Wolcott-Rallison Syndrome	2 (9)
Down's syndrome	2 (9)
Other chromosomal abnormality	2 (9)
Ehlers-Danlos syndrome	1 (4)
Pierre Robin sequence	1 (4)
**Skeletal dysplasia**	8 (21)
Basilar invagination	6 (75)
Vertebral fusion	5 (38)
Vertebral hypoplasia	4 (38)
Platybasia	4 (50)
**Traumatic injury**	6 (16)
Road traffic accident	3 (50)
Fall from height	3 (50)
**Inflammatory**	1 (3)
Juvenile Rheumatoid arthritis	1 (100)
Level of lesion*	
Craniocervical Junction	35 (78)
Sub-axial cervical spine	10 (22)
Pre-operative Modified McCormick Scale score*	
1	14 (31)
2	13 (29)
3	5 (11)
4	8 (18)
5	5 (11)

Of the 45 procedures, combined SSEP and MEP IONM was successful in all but one case whose cervical fixation was unmonitorable following a road traffic accident which resulted in a severe C1/2 dislocation. The majority of procedures had at least one EP modality monitored using modified alert criteria (SSEPs: 22/45, 49 %; MEPs: 23/45, 51 %). Most alerts were detected via MEPs (11/19, 58 %) followed by SSEPs and MEPs in combination (7/19, 37 %).

**Fig. 1:**
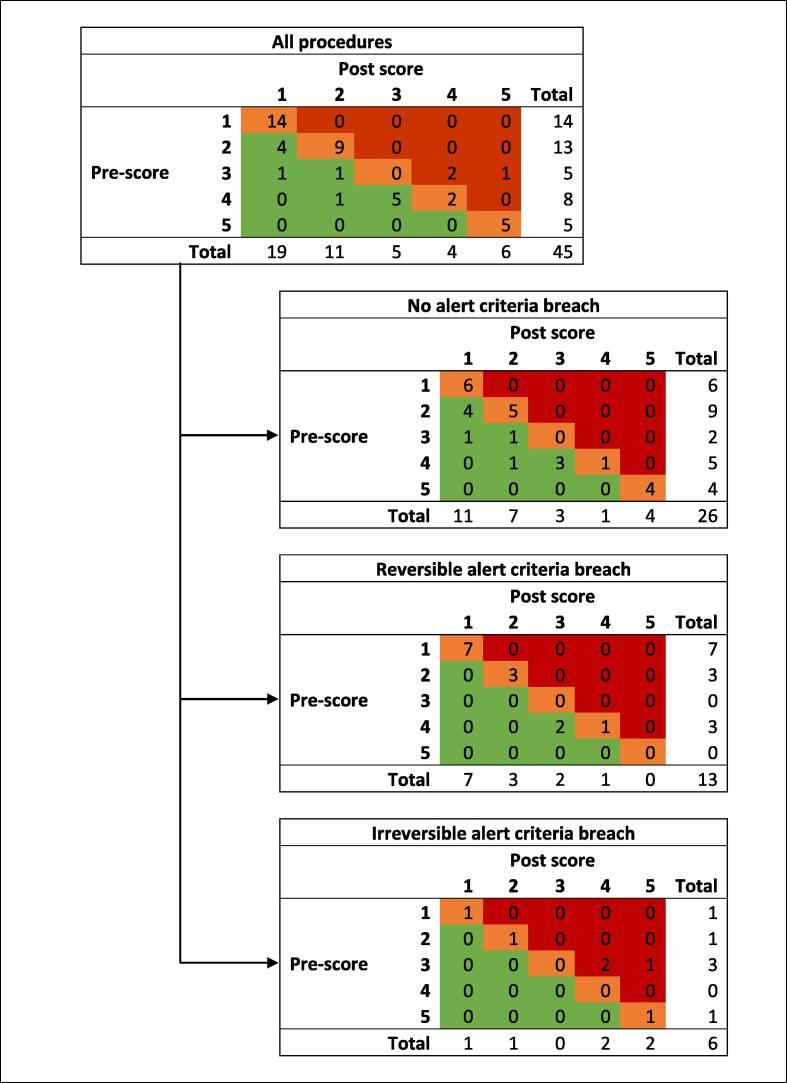
Pre- and post-Modified McCormick Scale score for all procedures and by alert breach type.

Alert criteria were breached in 19/45 (42 %) procedures during spinal column correction (3/19, 16 %), instrumentation (6/19, 32 %), hypotension (5/19, 26 %), and patient positioning (3/19, 16 %). Two breaches (11 %) had unknown causation (Table 3). Following the intervention protocol, EPs recovered to baseline in 13/19 (68 %). In 4/19 (21 %) procedures, EPs were at least 80 % lower in amplitude when compared to baseline. In 2/19 (11 %), EPs remained absent and both patients had worsening MMS postoperatively (Fig. 1).

**Table 3:** Demographics and IONM characteristics by type of alert breach. Number and percentage reported unless stated otherwise. MMS – Modified McCormick Score.

Characteristic	No alert criteria breach (N = 26)	Alert criteria breach		
		Overall breach (N = 19)	Reversiblebreach (N = 13)	Irreversible breach (N = 6)
**Age, years – mean (SD)**	8. (5)	9 (3)	9 (3)	9 (4)
**Female**	18 (69)	9 (47)	5 (38)	4 (67)
**Diagnosis**				
Congenital disease	16 (62)	13 (68)	10 (77)	3 (50)
Traumatic injury	5 (19)	3 (16)	2 (15)	1 (17)
Morphologic abnormalities	5 (19)	3 (16)	1 (8)	2 (33)
**SSEP baseline**				
Standard alert criteria	11 (42)	12 (63)	8 (62)	4 (67)
Modified alert criteria	15 (58)	7 (37)	5 (38)	2 (33)
**MEP baseline**				
Standard alert criteria	11 (42)	11 (58)	9 (69)	2 (33)
Modified alert criteria	15 (58)	8 (42)	4 (31)	4 (67)
**Alert detection modality**				
SSEP	NA	1 (5)	1 (8)	0
MEP	NA	11 (58)	9 (69)	2 (33)
Both	NA	7 (37)	3 (23)	4 (67)
**Alert causation**				
Correction	NA	3 (16)	1 (8)	2 (33)
Instrumentation	NA	6 (32)	5 (38)	1 (17)
Hypotension	NA	5 (26)	4 (31)	1 (17)
Positioning	NA	3 (16)	3 (23)	0
Unknown	NA	2 (11)	0 (0)	2 (33)
**Postoperative MMS**				
1	11 (42)	8 (42)	7 (54)	1 (17)
2	7 (27)	4 (22)	3 (23)	1 (17)
3	3 (12)	2 (11)	2 (16)	0
4	1 (4)	3 (16)	1 (8)	2 (33)
5	4 (15)	2 (11)	0	2 (33)

There was an association between the difference in pre- and post-MMS and the type of alert breach (p = 0.002). Deterioration in MMS was seen exclusively in irreversible alert criteria breach patients. Stable MMS was seen more frequently when alert criteria were not breached or when the breach was reversible. Improved MMS was seen more frequently in the no alert breach group (Table 4). There was no evidence of an association between type of breach (no alert breach, reversed breach, and irreversible breach) and diagnosis (p = 0.717); between the type of breach (irreversible, or reversible breach) and the alert detection modality (p = 0.200), causation of breach (p = 0.122) and postoperative MMS (p = 0.229).

**Table 4:** 3x3 Contingency table of postoperative MMS in patients with no, reversed, or irreversible alert criteria breach. MMS – Modified McCormick Scale score.

Type of breach	Difference from pre- to post-operative MMS			Total
	Improved	Stable	Worse	
No alert breach	10	16	0	26
Reversed breach	2	11	0	13
Irreversible breach	0	3	3	6
Total	12	30	3	45

## 4 Discussion

IONM was possible in 98 % of complex paediatric cervical spine fixation cases and criteria based on amplitude-reduction detected all postoperative worsening of MMS. The rate of alert criteria breach was high and frequently reversed following an intervention protocol. The chance of worsening neurological function was high if the breach was irreversible.

The majority of procedures were correction of atlantoaxial subluxation, instability, with or without spinal cord compression. This high-risk procedure is associated with postoperative neurological injury, especially in congenital diseases such as Mucopolysaccharidosis (MPS) (Solanki et al., 2013, Pauchard et al., 2014, Charrow et al., 2015). MPS patients made up 32 % of alert criteria breaches and 33 % of irreversible breaches with worsening MMS. Previous studies in paediatric CCJ surgery report between 10 and 20 % of cases breaching IONM alert criteria (Karandikar et al., 2012, Kim et al., 2015), which is lower than our cohort. These studies combined only include one MPS patient and it is difficult to delineate whether amplitude reduction criteria were utilised, and the details of the post-breach intervention protocol. With these exceptions in mind, our findings are comparable to the sparse literature in this field and may explain the high rate of breach reporting.

The majority of breaches reversed following the implementation of an intervention protocol. This is similar to other groups who report intervention protocol efficacy (Acharya et al., 2017, Zuccaro et al., 2017, Mohammad et al., 2018). Reversed breaches were identified more frequently in the instrumentation, hypotension, and positioning categories of alert causation, in comparison to when causation was unknown which is similar to existing literature (Holdefer and Skinner, 2020).

There were three patients who irreversibly breached amplitude reduction criteria and did not develop postoperative sensorimotor deficit. One occurred following a sustained period of haemodynamic instability during cervical spine fixation following a road traffic accident. This coincided with left deltoid, then global MEP breach. Following our intervention protocol, all MEPs recovered with the exception of the left deltoid muscle which remained at 19 % of its baseline amplitude. The second occurred during cervical column distraction to realign and correct C1/2 rotatory subluxation. During placement of the left sided rod, left sided MEPs breached alert criteria. On removing the rod, they returned but intermittently breached 80 % alert criteria. All breached MEPs reversed except the left tibialis anterior muscle (15 % of baseline amplitude). The patient was discharged with no new neurological deficits. We believe that any residual/subtle neurological deficit recovered by the time the patient woke up, and certainly within our 3-month postoperative assessment, thus explaining the lack of postoperative deficit in both cases. The third occurred during cervical vertebrae distraction and rod placement for a patient undergoing occipitocervical fixation. Upper limb SSEPs breached 50 % alert criteria bilaterally, followed by right deltoid MEPs. An adjustment to the correction and an increase in blood pressure reversed the SSEP alert, although right deltoid MEPs remained low amplitude, approximately 20 % of baseline. Postoperatively the child developed a right C5 palsy which was resolving at 3-month follow-up, and right *C*2 numbness. He was an MMS grade 2 preoperatively and postoperatively.

Our definition of a reversible alert was recovery in MEP to > 20 % of the baseline value. This was based on widespread clinical practice and the IONM literature (Langeloo et al, 2007), although these observations suggest a smaller recovery in MEP amplitude may still be associated with good or stable neurological outcome.

Alerts were more frequently identified in the MEP modality, perhaps due to increased sensitivity in detecting spinal cord ischaemia when compared to SSEPs (Shils and Deletis, 2020). However, inter- and intra-individual variability of muscle MEPs under prolonged general anaesthesia can make amplitude-reduction criteria vulnerable to false reporting (Ugawa et al, 2018). Eighty percent reduction alert criteria used in spinal surgery have 91–100 % sensitivity, 91–100 % specificity; a 61–100 % positive predictive value, and a 76–100 % negative predictive value (Journée et al, 2017). Measures of diagnostic accuracy were not calculated as estimates may be unreliable in this small sample. Although appropriate steps were taken to reduce false reporting, we cannot completely exclude this as a contributing factor to the high rate of reported alerts.

This study has several limitations. Firstly, its retrospective nature in evaluating MMS introduces an element of bias which affects the quality of evidence. We attempted to reduce this by blinding the MMS interpreter to IONM data. It is striking that there were no patients with either no alert criteria breach or reversed alert breach with worse outcome postoperatively, suggesting this to be a true finding. Secondly, whilst the MMS is validated for patients with spinal cord injury, it is less reliable in children who may not be able to communicate subtle deficits. Our assessment of sensorimotor function in studies moving forward will be more detailed and paediatric focussed. Finally, the small sample size will affect the strength of our findings.

## 5 Conclusion

IONM of SSEPs and MEPs during complex paediatric cervical spine surgery is feasible and detects neurological injury with a high degree of accuracy. When amplitude-reduction alert criteria are breached, an intervention protocol which re-established EPs never resulted in worsening of postoperative sensorimotor function.

## CRediT authorship contribution statement

**William M. McDevitt:** Conceptualization, Methodology, Investigation, Resources, Data curation, Writing – original draft, Visualization, Project administration. **Laura Quinn:** Formal analysis, Data curation, Writing – review & editing. **W.S.B. Wimalachandra:** Investigation, Data curation, Writing – review & editing. **Edmund Carver:** Writing – review & editing. **Catalina Stendall:** Writing – review & editing. **Guirish A. Solanki:** Writing – review & editing. **Andrew Lawley:** Conceptualization, Methodology, Writing – review & editing, Supervision, Project administration.

## Declaration of Competing Interest

The authors declare that they have no known competing financial interests or personal relationships that could have appeared to influence the work reported in this paper. The authors disclose receipt of the following financial support for the publication of this article: William McDevitt is supported by the NMAHPs Integrated Clinical Academic Research Unit, Birmingham Health Partners, UK.
