# Supplementary material for: Amplitude-reduction alert criteria and intervention during complex paediatric cervical spine surgery
Source: Clin Neurophysiol Pract. 2022 Jul 28;7:239–44. doi: 10.1016/j.cnp.2022.07.003 (PMC9420322; doi:10.1016/j.cnp.2022.07.003)
Supplement: Supplementary Data 1 [file mmc1.docx]

**IONM protocol**

**Abbreviations**

UL: Upper limb; LL: Lower limb; SSEP: Somatosensory evoked potential; MAC: minimal alveolar concentration; TIVA: total intravenous anaesthesia

**Generic guidelines**

- Recording electrodes impedance should be <5KOhms and equal.
- Recording electrodes used are likely to be corkscrew needle electrodes as they provide low resistance and avoid resistance changes over long periods of time (Davis and Kaye, 2020). However, disk or adhesive electrodes may be used as an alternative, when required, with adequate skin preparation.
- Bunch or twist leads together to reduce noise interference.
- Aim to avoid the application of notch filters.

**Stimulation Parameters: SSEP (Table 1)**

| **Stimulus** type | Rectangular constant current pulse |
| --- | --- |
| **Duration** | 0.2 – 0.3ms |
| **Intensity** | Determined by train of four technique to ensure supra-maximal response and ensure no neuromuscular block. |
| **Site** – UL: Median nerve  Note: *consider median unless surgical field incorporates, or is below C6-C7, or if cannulas prevent bilateral median nerve stimulation.*  *For ulnar stimulation, move anode and cathode to ulnar aspect of wrist.* | - Anode position – between flexor carpi radialis and palmaris longus tendons at the wrist crease - Cathode position – 3cm proximal to anode |
| **Site** – LL: Posterior tibial nerve | - Anode position – 3cm distal to cathode      - Cathode position – between the medial malleolus and Achilles tendon |

*Table 1: stimulation parameters.*

**Recording parameters: SSEP (Table 2)**

Technical aspects

- Filters
  - Low: 30Hz- this enhances scalp SSEP reproducibility but may distort peripheral potentials. Consider 0.2Hz LFF for peripheral recordings.
  - High: 300Hz for cortical, 1000Hz for peripheral.
- Rate: 4.7 – 5.1Hz. Consider lowering in those under two years or for lower limb stimulation.
- Averages: not predefined. Make sure number of averages optimises reproducibility to high-medium level
- Sampling rate: twice the high frequency filter setting (this prevents waveform aliasing). 16 bit 3-4KHz sampling rate is recommended.

|  | Peripheral responses | Segmental potentials | Sub-cortical potentials | Cortical potentials |
| --- | --- | --- | --- | --- |
| **UL** |  | | | |
| Location | Erb’s point (2cm above the mid-point of the clavicle) | C6/7 | C3’ or C4’- Must be ipsilateral to site of stimulation | C3’ or C4’ (2cm posterior to C3/C4) - must be contralateral to site of stimulation |
| Electrode | Surface electrode | Surface electrode | Corkscrew | Corkscrew |
| Reference | Fz | Fz and local reference for spine/if recording channel is available | Erb’s point (contralateral to stimulation site) | Ipsilateral cortex (C3’ and C4’) **AND** Fz |
| Rationale | Ensure nerve is stimulated, acts as a localiser if cortical potential is absent, can monitor brachial plexus palsy. | Acts as a localizer. | Acts as a localiser for potentially injured zone and depth of anaesthesia. | Main determinant of spinal cord/cortical injury. |
| **LL** |  | | | |
| Location | Popliteal fossa (just superior to the crease of the fossa) | T12/L1/Conus | Fz | Cz’ (2cm posterior to Cz) |
| Electrode | Surface electrode | Surface electrode | Corkscrew | Corkscrew |
| Reference | Local (2-3cm above active) | Fz. Consider local reference if recording channel is available | Erb’s or Cv7 | Contralateral cortex (C3’ or C4’, opposite side to site of stimulation) **AND** Fz |
| Rationale | Ensure nerve is stimulated, acts as a localiser if cortical potential is absent | Acts as a localiser | Acts as a localiser for potentially injured zone and depth of anaesthesia. | Main determinant of spinal cord/cortical injury. |

*Table 2: recording parameters for peripheral responses, segmental potentials, sub-cortical potentials and cortical potentials.*

*Note: If Erb’s point cannot be recorded, place electrode at the cubital fossa, medial to the bicep tendon just superior to the fossa crease. NB: Brachial plexus palsies may be missed.*

*Note: Subcortical potentials are robust during anaesthesia which includes inhaled agents, so may be useful to identify lack of cortical SSEP cause due to anaesthesia. However, they (usually) require 500-1000 averages and might not be useful when trying to optimise SSEPs. Only use in conjunction with cervical spine segmental recording.*

**Anaesthesia**

- Premedication (sedation, analgesia) can have an effect on EPs but this is usually minimal by the time intraoperative baselines are acquired.
- If inhaled agents (for example Isoflurane with Nitrous Oxide) are unavoidable, limit their use to induction.
  - <0.5 MAC halogenated gas with reduced Propofol may be acceptable.
- Total intravenous Remifentanil and Propofol is recommended for optimum SSEP recording.
- Ketamine or Etomidate have minimal effect but should not be given as bolus.

**Interpretation**

Reproducibility

| Reproducibility | Amplitude variation | Waveform superimposition | Detectable decrement |
| --- | --- | --- | --- |
| High | <20% | Nearly exact | >~30% |
| Moderate | 20-30% | Approximate | >~40% |
| Low | 30-50% | Loose | >~50% |
| Not-reproducible | >50% | Divergent | Disappearance |

*Table 3: Reproducibility classification and detectable pathological decrements [MacDonald et al, 2019].*

Warning criteria

>50% decrease in cortical amplitude after thorough interpretation of SSEP reproducibility (refer to table 3). Consider all variables in Table 3 before deciding on what warning criteria to use. A comment pertaining to SSEP reproducibility should be documented in annotations.

Before communication of an alert make sure:

- Blood pressure is optimal and has not changed recently;
- No boluses of anaesthesia have been given;
- The patient is not hypothermic (this usually prolongs latency but can affect amplitude);
- Scalp oedema from fluid boluses may decrease amplitude. Rule this out;
- Brachial or lumbar plexus palsy (limb positioning);
- Patient positioning (may increase blood pressure);
- Take into account the reproducibility of your responses (table 3);
- Have MEPs also deteriorated?

When communicating an alert:

- Be clear;
- Offer a rationale for deterioration;
- Provide strategy to reverse alert.

Setting baselines

Benign systemic influences can cause a baseline drift in amplitude so don’t set an “early” baseline. Take everything into account when calling an alert. You may need to reset baseline.

Downward drift

This refers to systemic factors (patient temperature, anaesthesia, blood pH, electrolyte imbalance) which alter SSEP amplitude and latency. For example:

- a drug bolus may increase latency and decrease amplitude;
- switching from inhaled agents (during induction) to TIVA (for maintenance) may increase amplitude and decrease latency;
- Decreased/increased patient temperate will increase/decrease onset latency but have very little effect on amplitude (unless hypothermic).

If you suspect downward drift following IONM break:

- Discuss findings with anaesthetist and confirm:
  - No change in temperature;
  - No anaesthetic bolus/change;
  - Correct pH and electrolyte blood levels.

If thought to be due to systemic factors, proceed with operation and work with anaesthetist to optimise SSEPs. If though to be directly related to surgery or perfusion, treat it as an alert.

**Indications for SSEPs**

- Peri-Rolandic brain surgery.
  - Phase reversal.
- Cerebrovascular surgery (intracranial aneurysm, AVMs and endarterectomy
  - Prevent sensory cortex ischaemia.
- Posterior fossa surgery.
  - Monitor integrity of medial lemniscus tract.
- Spinal deformity.
  - Assessment of dorsal columns.
- Spinal neurosurgery.
  - Midline mapping.
- Descending aortic procedures.
  - Monitor spinal cord blood flow.

**SSEP optimisation**

SSEP optimisation recommendations from MacDonald *et al*. See “SSEP optimisation” protocol for detail. Essentially, these documents can be considered before each operation and may be more useful in situations where preoperative SSEPs are difficult to record.

**REFERENCES**

Davis, SF and Kaye, AD. Principles of Neurophysiological Assessment, Mapping and Monitoring, second edition. Chapter 6: Somatosensory-evoked potential monitoring; 2020, pp 99 – 111.

Grover H, Walsh P, Sanders B, Shirley C. Updated ANS/BSCN guidelines for neurophysiological recordings of the spinal cord during corrective spinal deformity surgery; 2018.

MacDonald DB, Dong C, Quatrale R, Sala F, Skinner S, Soto F, Szelenyi A. Recommendations of the International Society of Intraoperative Neurophysiology for intraoperative somatosensory evoked potentials. *Clinical Neurophysiology.* 2019; **130**: 161-179.

**MEPs**

Abbreviations: UL: Upper limb; LL: Lower limb; MEP: Motor evoked potential; LMN: Lower motor neuron; CST: Corticospinal tract; ISI: Inter stimulus interval; p/s: pulses per second; NMJ: Neuromuscular junction; MAP: Mean arterial pressure; DCS: Direct cortical stimulation; IMSCT/EMSCT: Intra- and Extramedullary spinal cord tumour; LFF: Low frequency filter; HFF: High frequency filter

**Safety**

It’s now generally considered that MEPs are a safe technique to employ during IONM. Safety issues reported include:

- Seizures.
  - Propofol infusion should protect against this but seizure risk is more likely if patient is epileptic and/or during brain surgery.
- Bite injury
  - Rare if bite blocks are correctly positioned.
- Movement induced injury.
- Arrhythmia.
  - Monitored by anaesthetists.

The high charge required to elicit MEPs could be toxic if applied directly to cortical tissue but the scalp disperses the charge by a factor of 1:20. The maximum published MEP parameters produce charge and charge density below the experimental injury threshold, therefore MEPs are unlikely to cause excitotoxicity.

However, as a precaution the following conditions may warrant further explanation of MEP stimulation risks during the informed consent process:

- Epilepsy.
  - Increased risk of seizure.
- Skull defect
  - Dissipated charge, via skull, may “leak” into defect.
- Intravascular clips, shunts, electrodes
  - Small risk of causing haemorrhage, traumatic or infectious complication.
- Pacemakers/implanted bioelectric device
  - Risk of stimulation causing device to fail.

**Stimulation parameters**

Electrodes: Corkscrew. Consider disposable surface electrodes at the request of Anaesthetist or surgeon; or if the patient is a neonate. Rationale for surface electrode use must be documented in IONM report.

- When to stimulate
  - 10–15-minute intervals between other modalities.
  - Following prolonged neurotonic/myokymic discharges.
  - As required by surgeon.

Electrode sites

- M1/M2
- M3/M4
- Mz

Can also consider C electrode sites.

Montage: M1-M2/M2-M1. Can consider:

- M3-M4/M4-M3 to preferentially elicit UL MEPs
- M1,M2,M3,M4-Mz if cortical ischaemia is a risk factor for surgery
  - Consider for supratentorial tumours and vascular surgery.

**Trains**

Myogenic MEPs are high in amplitude and do not require signal averaging; signal averaging should in fact be avoided as MEPs often display a large amount of trial-to-trial variability. Instead, a train of stimuli causes synaptic transmission at the anterior horn cell and facilitates the production of I-waves which increases the train potency.

At least 3 pulses are required to evoke a muscle MEP. Adding pulses reduces MEP threshold and increases amplitude, duration and polyphasia. More pulses may be needed to elicit LL MEPs.

The ideal number of trains will vary between patients, but between 5-7 may be optimal and a priming pulse (train of 2) delivered before main train could help reduce trial-to-trial variability.

Each stimulus train activates a small fraction of anterior horn cells; a varying subset of the lower motor neuron pool is recruited with each run, causing variability in the response waveforms between each run.

**ISI (refer to generic guidelines for ISI conversion to p/s).**

a 2ms ISI (500p/s) is within the D-wave refractory period. Using a 4ms ISI (250p/s) allows full D-wave recovery, which is why MEP p/s should be set to 250 but could also acquire good MEPs at 3ms ISI.

Consider an ISI between 1-2ms (500-1000p/s) if you are only interested, or preferentially want to record hand muscle MEPs. This may be useful if recording corticobulbar MEPs in conjunction, as this ISI duration helps separate CoMEPs from stimulus artefact.

**Duration**

50-75μs. In some circumstances increasing the pulse duration to 1ms may increase MEP amplitude.

**Facilitation**

This acts as a preconditioning stimulus to raise excitability of corticomotor neurons. You can consider double-train stimulation to increase MEP amplitude at short inter train intervals (10-20ms). You can set double train facilitation at a 1-2Hz rate of acquisition (be mindful of patient movement).

Facilitation of lower motor neurons can also work. Just before eliciting MEP run a train of four on the limb muscles desired to elicit MEPs from.

**Recording parameters**

- LFF: 10-100Hz.
  - May prevent cranial nerve MEP recording.
    - Consider opening LFF to 0.2-2Hz.
- HFF: 1500 – 3000Hz.
  - May prevent cranial nerve MEP recording.
    - Consider restricting HFF to 150-300Hz.
- Timebase: Start at 100ms and adjust as appropriate.
  - Shorter timebase may be required for cranial nerve MEPs.
  - Longer time base if pathology suggests decreased conduction velocity.

Electrodes: Subdermal needles placed in the muscle of interest. If possible, place the active (negative electrode) over the belly of the muscle and the reference (positive) near the tendon. NB. Do not do this if it results in electrodes being too far apart.

Electrodes sites: This is surgery dependant. For the majority of cases aim to have X2 muscles sampled below site of surgery in both limbs and at least 1 muscle sampled bilaterally above the site of surgery (this acts as a control). Cases which may not follow this criterion:

- Brachial plexus lesion- only sample muscles of interest.
- Supratentorial, infratentorial and craniocervical junction surgery.
  - May be impossible to monitor MEP above site of surgery.
  - Control MEP can come from limb ipsilateral to surgical site.

The depth of needle insertion is at the discretion of the practitioner, however, keep the following in mind:

- A large amount of subcutaneous fat will place needle further from generator.
- AH and TA muscles are not typically surrounded by large amounts of subcutaneous fat.

**Anaesthesia**

Myogenic MEPs are highly sensitive to anaesthesia due to the summation of effects upon the synapse at the anterior horn cell. Therefore, avoid halogenated gas and NMJ blocking agents. Intravenous Propofol causes less suppression of LMN excitability in comparison to inhaled agents, which makes MEP monitoring more consistent (less trial-to-trial variation). Can consider etomidate, ketamine and benzodiazepine supplement to anaesthetic regime if the depth of anaesthesia is thought to be contributing to poor MEP results. Norepinephrine has a powerful effect on MEP excitability too.

**Blood pressure**

Brain and spinal cord autoregulation of blood flow should remain constant over a wide range of MAP (50-120mmHg); but this data is derived from adults and does not take into account the effect anaesthetic has on autoregulation and variability between subjects. Therefore, the lower limit of autoregulation may be anywhere between 33-113mmHg; which is why some advise keeping MAP at or above 70mmHg (age-dependant) to ensure adequate perfusion.

Acute spinal cord compression can produce transient hypertension due to a surge in noradrenaline, adrenaline and/or catecholamines which may also transiently increase MEP amplitude above the level of compression.

Local autoregulation can be disrupted by the patient’s pathology (tumour, lipoma) and surgical intervention (patty placement, pressure on spinal cord from retractor, distraction, traction). This must be kept in mind when interpreting focal MEP changes.

Changes in MEPs as a result of dysautoregulation are usually generalised but can be focal if local interruption to perfusion is presumed. The best response to this is to raise blood pressure.

NB: Keep in mind that an antihypertensive administered to control hypertension will also decrease amplitude of MEP whereas phenylephrine increases motor neuron excitability and may increase MEP amplitude. Magnesium sulphate reduces blood pressure and might reduce MEP amplitude by acting as a neuromuscular blockade.

NB: Raising blood pressure in response to MEP change can delay correct intervention in cases where the presumed cause of MEP decrease is surgical manoeuvre/manipulation.

**Interpretation**

*Trial-to-trial variability*

This is almost to be expected, and is attributed to fluctuating lower motor neuron background facilitation from upper motor neurons, propriospinal, and sensory synapses. The excitatory potentials need to summate and exceed the firing threshold before an MEP can be elicited: this is an on-off phenomena/all-or-nothing response. So, it is important to bare this in mind when trying to elicit an MEP. It only takes a small decrease in CST tract “drive” or decrease lower motor neurone excitability to produce a large reduction/absent MEP. This makes MEPs highly sensitive to change (due to systemic factors or spinal cord injury) but an imperfect predictor of motor deficit severity or permanence.

*MEP “fade”*

Despite “stable” anaesthesia, muscle MEPs may decrease in amplitude as the operation progresses, most likely due to decrease lower motor neurone excitability. This requires facilitation or increased stimulation intensity.

NB: Increasing stimulation intensity makes it problematic to accurately use amplitude-reduction criteria.

*Temperature*

Only deep hypothermia significantly impacts on MEP amplitude. Moderate change in temperature is more likely to increase/decrease onset latency.

Consider electrolyte disturbances, hypercapnia, hypocapnia or anaemia as less common causes of MEP alterations.

*Localisation*

Multiple muscles should be sampled per limb in order to be more specific about the potential site of injury. For example:

- Deltoid MEP preservation with HAND loss places the injury below C5/C6 and above or at C8/T1

Criteria for alert (Table 4)

The criteria used to alert the team is surgery specific and will depend on the level of sensitivity deemed appropriate, and the acceptability of false positive rates within the team. Please refer to specific IONM alert criteria protocol for more detail, but in brief, the following alert criteria can be applied to the surgeries listed in table 4.

| Surgery | Muscle MEP criteria | | | |
| --- | --- | --- | --- | --- |
|  | Absent | >50% decrease | >80% decrease | >100V threshold elevation |
| Supratentorial | Major | Major |  |  |
| Infratentorial | Major | Major |  |  |
| Facial nerve | Major | Major |  |  |
| IMSCT  (with D wave) | Major |  | Minor | Minor |
| Spine | Major |  | Moderate | Moderate |
| Aortic | Major |  | Moderate |  |
| Nerve root | Major |  |  |  |

*Table 4: Surgery-specific motor evoked potential alert criteria. Adapted and modified from ACNS warning criteria/MacDonald, 2017.*

**Major**: Always prompt pause in surgery and alert criteria breach protocol followed.

**Moderate**: Discuss findings with Neurosurgery and Anaesthetics before following alert criteria breach protocol.

**Minor**: Surgeon alerted and takes decision to follow alert criteria breach protocol.

*Indication for monitoring MEPs*

SSEP monitoring in isolation can miss selective injury to anterior horn cells, either as a direct result of surgical manipulation/procedure, restricted anterior artery blood flow or poor anterior horn cell perfusion. MEPs should be used in conjunction with SSEPs during:

- Supratentorial.
  - Tumour resection.
  - AVM resection.
- Infratentorial.
  - Tumour resection.
- Vascular surgery.
  - Anything with risk of reduced cerebral blood flow via carotid system.
- Spine
  - Intradural IMSCT, EMSCT.
  - Deformity correction (ACDF, scoliosis etc).
  - Trauma.
  - Spinal cord decompression.
  - Embolisation
    - Vascular tumour.
    - AVM.
  - Thoracoabdominal aneurysm repair.
- Peripheral nerve surgery.
  - Brachial plexus.
  - Parotid gland tumour.

**MEP Optimisation**

- Recording and referencing from adjacent muscles (i.e., Bicep/Tricep, ABP/ADM) can increase the amplitude of MEPs but is less specific to individual root/spinal level injury. For example, monitoring a C7/C8 ACDF surgery with Bicep-tricep MEP may not be sensitive enough to detect a lesion at C7/C8. ABP/ADM are an exception to the above as they are both innervated by the same roots, so you can opt for this “HAND” recording style.
- Consider facilitation section

**References**

Davis SF, Kaye AD, editors. Principles of neurophysiological assessment, mapping, and monitoring. Springer International Publishing; 2020.

MacDonald DB, Skinner S, Shils J, Yingling C. Intraoperative motor evoked potential monitoring- A position statement by the American Society of Neurophysiological Monitoring. *Clinical Neurophysiology.* 2013;**124:** 2291-2316

MacDonald DB. Overview on criteria for MEP monitoring. *Journal of Clinical Neurophysiology.* 2017;**34**: 4-11

MacDonald DB, Zayed ZA, Al Saddigi A. Four-limb muscle motor evoked potential and optimised somatosensory evoked potential monitoring with decussation assessment: results in 206 thoracolumbar spine surgeries. *European Spine Journal.* 2007;**16**(Suppl 2):S171-S187.

Simon M. Intraoperative Neurophysiology. A comprehensive guide to monitoring and mapping. *Demos Medical*, New York.
